# Neonatal body composition by air displacement plethysmography in healthy term singletons: a systematic review

**DOI:** 10.1186/s12887-019-1867-y

**Published:** 2019-12-12

**Authors:** Cornelia Wiechers, Sara Kirchhof, Christoph Maas, Christian F. Poets, Axel R. Franz

**Affiliations:** 1 Department of Neonatology, University Children’s Hospital, Eberhard Karls University, Tuebingen, Calwerstr. 7, 72076 Tuebingen, Germany; 2 Center for Pediatric Clinical Studies, University Children’s Hospital, Eberhard Karls University, Tuebingen, Germany

**Keywords:** Infant, Neonatal, Body composition, Air displacement plethysmography, Fat mass

## Abstract

**Background:**

There is increasing evidence that intrauterine environment and, consequently, growth in utero have both immediate and far-reaching consequences for health. Neonatal body composition might be a more sensitive marker of intrauterine environment and neonatal adiposity than birth weight and could serve as a predictor for non-communicable diseases later in life.

**Methods:**

To perform a systematic literature review on neonatal body composition determined by air displacement plethysmography in healthy infants. The systematic review was performed using the search terms “air displacement plethysmography”, “infant” and “newborn” in Pubmed. Data are displayed as mean (Standard deviation).

**Results:**

Fourteen studies (including *n* = 6231 infants) using air displacement plethysmography fulfilled inclusion criteria for meta-analysis. In these, weighted mean body fat percentage was 10.0 (4.1) % and weighted mean fat free mass was 2883 (356) g in healthy term infants. Female infants had a higher body fat percentage (11.1 (4.1) % vs. 9.6 (4.0) %) and lower fat free mass (2827 (316) g vs. 2979 (344) g). In the Caucasian subpopulation (*n* = 2202 infants) mean body fat percentage was 10.8 (4.1), whereas data for reference values of other ethnic groups are still sparse.

**Conclusions:**

Body composition varies depending on gender and ethnicity. These aggregated data may serve as reference for body composition in healthy, term, singletons at least for the Caucasian subpopulation.

## Background

There is strong evidence that fetal and early postnatal environment play an important role in fetal programming and in determining the risk for disease in adulthood such as obesity, diabetes and cardiovascular disease [1, 2]. Proposed key underlying mechanisms include epigenetic influences on DNA expression, the intrauterine development of hormonal axes and the relative accretion of different tissues and body components [3]. Body composition at birth may serve as a surrogate marker for the environment in-utero [4].

Fetal growth and body composition at birth are influenced by numerous factors, some non-modifiable such as gender, gestational age and ethnic/genetic background, others modifiable such as maternal diet as well as weight gain and (metabolic) health during pregnancy. These modifiable prenatal factors may affect the health of the offspring throughout his/her entire life [5, 6].

In a recently published meta-analysis including 477,620 children aged 2 to 13 years in Europe, the pooled prevalence of overweight and obesity ranged from 13 to 23% in various regions in the period between 2011 and 2016 [7]. In a recent study on children and adolescents aged 2 to 19 years in the United States, the prevalence of obesity was 17.0% in 2011–2014 and that of extreme obesity was 5.8% [8], indicating the increasing relevance of childhood obesity for public health. Obesity in children is associated with elevated blood pressure and abnormal fasting glucose concentrations [9]. Furthermore, obese children are likely to become obese adults with an increased risk of obesity-related complications (e.g. diabetes and cardiovascular disease) and increased morbidity and mortality [10–12].

Neonatal body composition parameters such as fat mass (FM), fat free mass (FFM) and the proportion of FM divided by total body mass (BF%), might be more sensitive markers of the in-utero environment and neonatal adiposity than birth weight and length alone, because variability in FM and FFM has been reported in newborns of similar weight and length [13, 14], and anthropometric measures, albeit easy to determine, do not necessarily reflect variability in body composition.

Neonatal body composition can be determined by skinfold thickness, isotope dilution, dual energy x-ray absorptiometry (DXA), magnetic resonance imaging (MRI) and air displacement plethysmography (ADP), where the latter was shown to produce highly reproducible and accurate measurements and may be suitable for large epidemiological studies [4, 15]. Furthermore, ADP has the advantages of not using ionizing radiation, a short examination time and comparatively low costs, hence ADP will likely be the method of choice for future studies.

To inform future studies, we performed a systematic review of the literature and meta-analysis for available measurements of body composition at birth in healthy, term, singleton infants using ADP. Our aim was to establish reference values for different ethnic groups and to investigate factors potentially influencing body composition.

## Methods

### Searches and information sources

An all-language literature search was carried out on September 14, 2018 in Pubmed using the search strategy ((“air displacement plethysmography”[All Fields]) AND (“infant, newborn”[MeSH Terms] OR (“infant”[All Fields] AND “newborn”[All Fields]) OR “newborn infant”[All Fields] OR (“infant”[All Fields] AND “newborn”[All Fields]) OR “infant, newborn”[All Fields]). This was complemented by an online list of references provided by the manufacturer of the ADP device (downloaded on November 22, 2018; latest update March 23, 2019; https://www.cosmed.com/images/pdf/bibliography/PEA_POD_Bibliography.pdf).

### Inclusion criteria

First, abstracts were screened for relevance and all articles reporting measures of body composition determined by ADP in full-term infants (≥ 37 0/7 SSW) during the first 96 postnatal hours selected. If investigators published more than one report on the same study cohort or several reports with overlapping populations, only the most recent publication or that with the most representative population was included. As we aimed for reference data, reports on less than 100 infants were excluded because such small populations were deemed potentially non-representative. Data on body composition (BF%, FM, FFM), anthropometry and birth details were extracted from relevant full-text articles. This process war carried out by two of the authors (C.W., S.K.).

### Statistical analyses

Observational data was pooled calculating weighted mean and standard deviation assuming that the summarized populations are samples of a single overall population (i.e., of healthy term singleton neonates). Between group comparisons were done based on means, standard deviations and sample size using two-sided t-test or ANOVA and post hoc Tukey’s multiple comparison test. The degree of variability of body composition of healthy singletons was assessed by calculating the coefficient of variation (coefficient of variation = (standard deviation / mean) * 100). Analyses were performed with GraphPad Prism® 8.1.0 (GraphPad Software, San Diego, CA, USA) and the level of significance was *p* < 0.05.

## Results

### Search results

**Fig. 1:**
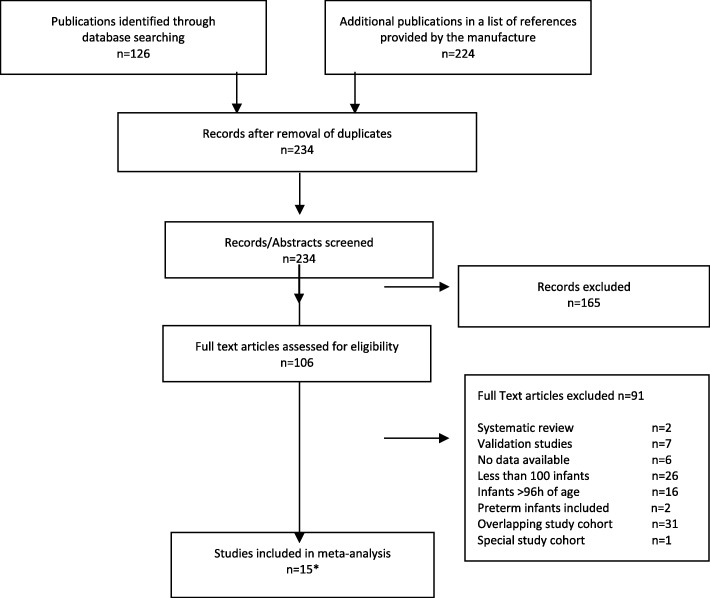
Flow chart of the systematic review process. * Grijalva-Eternod et al. [16] and Anderson et al. [17] reported on the same study population. Anderson et al. described differences in body composition between boys and girls, their report was therefore used for the evaluation of the association between gender and body composition Lee et al. [18] and Lampl et al. [19] also reported on the same study population, Lampl et al. reported in more detail on the ethnicity of the study population, their report was therefore used for the evaluation of the association of ethnicity and body composition.

Search results are detailed in Fig. 1. The initial search in Pubmed identified 126 publications. In the list of references on body composition provided by the manufacturer, we identified 224 publications. After removal of duplicates and screening of abstracts, 234 were discarded as it became clear from their abstracts or titles that they did not meet entry criteria or contained overlapping study cohorts. The remaining 106 citations were selected for full-text review of which 91 were excluded as detailed in Fig. 1.

The remaining 15 publications reported on 13 distinct populations. Two populations were reported twice, but with different aspects relevant to this review.

Additionally, data from a German Caucasian cohort recently studied at Tuebingen University Hospital was included as 14th population [20], resulting in a total of *n* = 6231 infants studied in Europe, Australia, Asia, North and South America, India and Africa. Characteristics of studies and participants as well as countries of origin are shown in Table 3.

### Study population

The (weighted) mean (SD) gestational age at birth was 39.6 (1.2) weeks and mean birth weight 3382 (456) g; mean age at assessment was 40.8 (23.1) h. The overall male/female ratio was 0.96.

### Body composition

In the healthy term singleton infants included in the 14 study populations (n = 6231), mean BF% was 10.0 (4.1) % and mean FFM 2883 (356) g (Table 1). Mean BF% ranged from 7.8% in Ethiopia to 13.6% in the USA. Analysis of body composition variability revealed a greater degree of variability for BF% than for FFM in all included infants (coefficient of variation for FFM was 12% whereas the CV for BF% was 42%).

**Table 1:** Characteristics of included publications, study populations and aggregate body composition data

Author, [Reference No]	Year of publication	Country	Total sample size, n	Limited to singletons	Ratio male/female	Mean age at assessment, h (SD)	Mean birth weight, g (SD)	Weight at assessment, g (SD)	Fat-free mass, g (SD)	Body fat mass/total body mass, % (SD)	Fat mass, g (SD)
Wiechers C et al. [20]	2019	Germany	271	Yes	0.77	44.4 (19.6)	3389 (440)	3200 (411)	2857 (330)	10.6 (4.0)	347 (157)
Systematic review											
Au CP et.al [4]	2013	Australia	599	Unknown	1.09	N/A	3417 (475)	3246^b^	2947 (342)	9.2 (4.4)	299^b^
Grijalva-Eternod CS et al. [16]	2015	Ethiopia	528	Unknown	0.96	N/A	N/A	3090 (380)	2840 (310)	7.8^b^	240 (150)
Hawkes CP et al. [21]	2016	Ireland	1063	Yes	1.02	44.2 [23]	3520^b^	3335^b^	2951^b^	11.1^b^	430^b^
Roggero P et al. [22]	2010	Italy	262	Yes	1.06	49.4^b^	3301 (308)	3091^b^	2794^b^	9.5^b^	297^b^
Brei J et al. [25]	2015	The Netherlands	194	Unknown	0.39	N/A	3384 (500)	3259 (490)	2916 (400)	10.3 (4.0)	345 (170)
Pereira-da-Silva L et al. [26]	2014	Portugal	100	Yes	0.82	50^b^	3360 (359)	3180^b^	2811 (250)	11.4 (4.1)	369 (156)
Tint MT et al. [24]	2016	Singapore	173	Yes	0.94	N/A	N/A	3123^b^	2770^b^	10.0^b^	313^b^
Lee W et al. [18]	2012	USA, Michigan	324	Yes	0.98	22.3^b^	3296 (560)	3304^b^	2967^b^	10.2 (4.0)	337 (173)
Paley C et al. [23]	2015	USA, New York	332	Yes	0.96	35.4^b^	3397^b^	3242^b^	2793^b^	13.6^b^	449^b^
Shapiro AL et al. [27]	2016	USA, Colorado	1079	Yes	1.04	N/A	3254^b^	3124^b^	2831^b^	9.0^b^	293^b^
Josefson JL et al. [28]	2016	USA, Chicago	168	Yes	1.05	N/A	3469 (499)	3267^b^	2900 (424)	10.8 (3.7)	367 (166)
Villar J et al. [29]	2017	Multicountry^c^	928	Unknown	N/A	N/A	N/A	3254 (509)	2906 (389)	10.3 (4.0)	348 (170)
Castro N P et al. [30]	2017	Brazil, Sao Paulo	210	Yes	0.84	N/A	3377 (408)	N/A	N/A	8.9 (4.2)^a^	N/A
Total, n			6231		5303	2352 (1334)^a^	4602 (2128)^a^	6021 (1921)^a^	6021 (2788)^a^	6231 (2794)^a^	6021 (2513)^a^
Weighted mean					0.96	40.8 (23.1)	3382 (455)	3218 (458)	2883 (356)	10.0 (4.1)	341 (164)

Not available N/A

^a^Weighted SD calculated from available SD values

^b^ SD not reported in original publication

^c^ Brazil, Ghana, India, Norway, Oman, USA

### Gender

Ten of the selected studies reported data separately for male and female infants (*n* = 3609; 1868 female). Meta-analysis showed that females had a higher mean BF% (11.1%) than males (9.6%; mean difference 2.0% (95%CI 1.7–2.3%; *p* < 0.0001)) (Table 2). Male infants had higher mean FFM (mean difference 152 g (95%CI 127-177 g; p < 0.0001)) as well as higher birth weight (mean difference 129 g (95%CI 88-170 g; p < 0.0001)).

**Table 2:** Gender and body composition

Gender	Reference-No.	Sample size, n	Birth weight, g (SD)	Weight at assessment, g (SD)	Fat-free mass, g (SD)	Body fat mass/total body mass, % (SD)	Fat mass, g (SD)
female	[4, 13, 18, 20, 21, 23, 25, 26, 28, 30]^a^	1868	3405 (438)	3196 (415)	2827 (316)	11.1 (4.1)	372 (169)
male	[4, 13, 18, 20, 21, 23, 25, 26, 28, 30]^b^	1741	3534 (443)	3324 (429)	2979 (344)	9.6 (4.0)	343 (164)

^a^ Own data contributed *n* = 153^b^ Own data contributed *n* = 118

Ref (13) contributed 3 pairs of twins in their sample (1.6% of children in that study)

### Ethnicity

In the eight studies [16, 18–24, 27] reporting ethnic background (*n* = 3203), we classified ethnic background into five different groups (Caucasian, African-American, Asian, Hispanics recruited in the USA, East-African). Meta-analysis showed that Hispanics recruited in the USA had the highest BF% with 14.3%, followed by African-American infants with 11.2% (Table 3). Lowest BF% was reported in East-African newborns (7.8%). FFM was highest in Caucasian infants followed by East-African and lowest in African-American. Lack of data on SD in the original reports precluded between-group comparisons by ANOVA.

**Table 3:** Ethnic background and body composition

Ethnic background	Reference-No.	Total sample Size, n	Fat-free mass, g (SD)	Body fat, % (SD)	Fat mass, g (SD)
Caucasian	[18–23]^b^	2202	2903 (363)	10.8 (4.1)	386 (168)
African-American	[18, 19, 23]	198	2675^a^	11.2^a^	342^a^
Asian	[23, 24]	203	2782^a^	10.3^a^	326^a^
Hispanics	[23]	72	2767^a^	14.3^a^	479^a^
East-African	[16]	528	2840 (310)	7.8^a^	240 (150)

^a^ SD not reported in original publication

^b^Own data contributed *n* = 271

### Age at assessment

Body composition according to postnatal age (in days after birth) at measurement was reported in four studies (*n* = 1051); mean postnatal age in these studies was 46.9 (6.8) h [18, 20, 22, 25]. Due to the study design [31], almost all East-African infants, who had remarkably low BF% compared to other populations as indicated in Table 3, had been examined on day 0. Since there was hardly any data on body composition for this subgroup on the following days, this subgroup was excluded from analysis of the association between postnatal age at assessment and body composition.

While body weight at assessment and FFM decreased with postnatal age at measurement and was lowest on postnatal day 3, (*p* < 0.0001 each), BF% and birth weights were similar in all four postnatal age groups (*p* = 0.63 and *p* = 0.10, respectively) (Table 4).

**Table 4:** Day of assessment and body composition

Postnatal age at assessment Days (hours) after birth assessment	Reference-No.	Total sample size, n	Birth weight, g (SD)	Weight at assessment, g (SD)	Fat-free mass, g (SD)	Body fat, % (SD)	Fat mass, g (SD)
D0 (0-24h)	[20, 22, 25]^a^	288	3382 (454)	3266 (442)	2922 (364)	10.3 (3.9)	346 (160)
D1 (25-48h)	[18, 20, 22]^a^	545	3332 (488)	3254 (368)	2923 (295)	10.1 (3.9)	331 (161)
D2 (48-72h)	[20, 22]^a^	132	3324 (382)	3086 (360)	2785 (303)	9.8 (3.7)	309 (141)
D3 (72-96h)	[20, 22]^a^	86	3247 (356)	3001 (312)	2688 (279)	10.3 (3.8)	313 (129)

^a^ Own data contributed *n* = 38 on D0, *n* = 125 on D1, *n* = 76 on D2, n = 32 on D3

## Discussion

Aim of this meta-analysis was to summarize and compare the currently available data on neonatal body composition in healthy term infants determined by ADP to inform future studies. In the studies selected for meta-analysis, median BF% was 10.0% (SD 4.1%) and mean FFM 2883 (356) g. German infants showed values for BF% (10.6%) [20] similar to those from other Europeean countries such as Portugal (11.3%) [26], the Netherlands (10.3%) [25], and Ireland (11.1%) [21], but higher values than those from Australia (BF% 9.2) [4] and the US (BF% 9–13.6%) [18, 23, 27, 28].

Meta-analysis of eight studies enabled comparisons of different ethnic backgrounds and showed that Hispanic infants recruited in the USA had the highest BF% with 14.3%, followed by African-American infants with 11.2%. The lowest BF% was reported for East-African newborns (7.8%). FFM was highest in Caucasian infants with 2903 (363) g, followed by East-Africans with 2840 (310) g and lowest in African-Americans (2674 (N/A) g).

Differences in total body fat between populations of different ethnic background have already been reported in adults and children [31, 32], but little data based on ADP exist in neonates. Paley et al. found a higher total FM in African–American, Asian and Hispanic males and African–American females compared to Caucasian males and females, respectively [23]. Furthermore, an Australian study reported that infants of Caucasian mothers showed higher BF% and birthweight compared to infants of Asian mothers [4]. In contrast, Ramel et al. measured body composition in preterm infants after hospital discharge in comparison to term infants and found no differences between „white “or „non-white “infants [33]. The data from this systematic review seem to support that there are differences in body composition between neonates from different ethnic backgrounds, but it remains unclear whether these are genetically determined or due to socioeconomic factors (e.g., access to nutrition etc.). Furthermore, the absolute differences in FFM and BF% reported herein must be interpreted with caution because lack of data on SD precluded statistical analyses by ANOVA and t-test.

In a cross-sectional Australian study including 599 term infants, gender showed the strongest association with neonatal BF%, followed by maternal ethnicity [4]. Consistent with this, the present study and meta-analysis confirmed differences in body composition between female and male neonates, with girls having a higher BF% (11.1% vs. 9.6%) and lower FFM (2827 g vs. 2979 g), which seems to remain true throughout life [34, 35]. Gender is known to be a major determinant of body composition for term infants: males are heavier at birth and have a higher lean body mass, whereas females have more subcutaneous fat [36]. Gender differences have been primarily attributed to the action of fetal sex steroid hormones, e.g. testosterone, which presumably enhances lean body mass growth in utero [37].

Besides ethnic factors and gender, the quality of maternal diet and intake of macro- and micronutrients during pregnancy as modifiable factors have demonstrated significant impact on birth outcomes including body composition [38, 39]. Therefore, maternal nutritional status during pregnancy is an important factor in fetal growth and development [27, 38–42] and changed over the last years in industrialized as well as developing countries. The Healthy Start study demonstrated the influence of poor diet quality during pregnancy on neonatal adiposity with increases in BF% but no differences in FFM [27]. It also reported that neonatal adiposity, but not birth weight, was independently associated with increased maternal intake of total fat and total carbohydrates [40], indicating that maternal diet is an important factor impacting on neonatal body composition but not birth weight.

Sparkes et al. hypothesized that fetal FFM is primarily influenced by genetic factors, whereas fetal FM is influenced by the maternal metabolic and nutritional environment [43]. This is consistent with our results indicating less variability in FFM compared to BF% across populations from industrialized countries. In the context of worldwide increasing public health challenges due to childhood obesity and later life metabolic dysregulation, interventions aiming at maternal nutritional exposure as well as maternal physical activity during pregnancy could be important [44].

Healthy newborn infants typically loose about 6–7% of their initial birth weight in the first days after birth [45, 46], and this weight loss is influenced by several factors (e.g., volume of feeding after birth, pre-delivery intravenous fluids, etc.). In a longitudinal study involving 28 exclusively breastfed, healthy, term infants during their first 5 postnatal days, Roggero et al. [22] showed that body composition changes with early postnatal weight loss and that both BF% and FFM decreased postnatally. However, there was a greater loss in BF% compared to FFM initially. In this meta-analysis summarizing cross-sectional data, FFM decreased along with body weight during the first 4 days after birth – whereas BF% differed little, indicating that FFM and FM are lost in similar proportions during the early postnatal weight loss. Admittedly, the longitudinal study of Roggero with repeated measurements in the same cohort is better suited to evaluate which compartments are affected by postnatal weight loss than this meta-analysis.

Limitations of our analysis are the limited number of studies and their heterogeneous design. Data on various influencing factors (e.g. age at measurement) were not published for all study populations. Nonetheless, body composition was measured in an objective and reproducible fashion using the same technique in healthy term (and predominantly singleton) neonates. The relatively homogeneous results found for body composition in our meta-analysis suggest good generalizability to other industrialized countries.

## Conclusions

Our systematic review revealed different body composition results for infants from different ethnic or socioeconomic backgrounds. Therefore, reference data for individual populations may be needed. Gender seems to affect not only body weight, but also body composition and thus also needs to be considered. Increasing postnatal age during the first 96 postnatal hours did not seem to affect BF% but was associated with decreased body weight at assessment and FFM.

## Data Availability

De-identified individual data will not be made available, because trial subjects have not been asked to consent.

## Abbreviations

ADP: Air displacement plethysmography
BF%: Proportion of fat mass/total body
BMI: Body mass index
FFM: Fat free mass
FM: Fat mass
N/A: Not available
SD: Standard deviation
SDS: Standard deviation score
